# The complete chloroplast genome of *Vanilla shenzhenica* (Orchidaceae)

**DOI:** 10.1080/23802359.2019.1642165

**Published:** 2019-07-17

**Authors:** Ting-Zhang Li, Li-Jun Chen, Meng Wang, Jian-Bing Chen, Jie Huang

**Affiliations:** a Key Laboratory of National Forestry and Grassland Administration for Orchid Conservation and Utilization, Shenzhen, China;; b Shenzhen Key Laboratory for Orchid Conservation and Utilization, The Orchid Conservation and Research Centre of Shenzhen, The National Orchid Conservation Centre of China, Shenzhen, China

**Keywords:** *Vanilla shenzhenica*, chloroplast genome, Orchidaceae, Shenzhen

## Abstract

*Vanilla shenzhenica* Z.J.Liu & S.C.Chen is a new species of orchid found in Shenzhen, South China for the first time. Here we report the complete chloroplast (cp) genome sequence and the features of* V. shenzhenica*. Its cp genome sequence was 151,537 bp, including one large single-copy region (LSC, 87,487 bp), one small single-copy region (SSC, 19,172 bp), and two inverted repeat regions (IRs, 22,439 bp). It encoded 123 genes, of which 104 were unique genes (69 protein-coding genes, 31 tRNAs and 4 rRNAs). The phylogenetic relationships show that *V. shenzhenica* is sister with *V. aphylla*.

The genus *Vanilla* (1754:4) was established by Plumier, and now approximately 70 species are recognized within the genus, and 4 species are distributed in China. Some new species of this genus have been published (Liu et al. 2007; Emerson et al. 2012; Francisco et al. 2014; Emerson and Marcelo 2016). *Vanilla* belongs to the subfamily Vanilloideae (Orchidaceae), and are distributed throughout the tropics (Chen et al. 2009; Pridgeon et al. 2009). It is charactered by climbing and fleshy stem, its fruit a pod (Tsi et al. 1999). *Vanilla* orchids are widely used as a cacao-beverage spice (Pesach, Kenneth, et al. 2008; Pesach, Severine, et al. 2008).

Leaf samples of *V. shenzhenica* were obtained from the Orchid Conservation and Research Centre of Shenzhen, and specimens were deposited in the National Orchid Conservation Center herbarium (NOCC; specimen code J.B.Chen 00012). Complete chloroplast genome sequence of *V. shenzhenica* was assembled in this study. Total genomic DNA was extracted from fresh material using the modified CTAB procedure of Doyle and Doyle (1987). Sequenced on Illumina Hiseq 2500 platform (Illumina, San Diego, CA). Genome sequences were screened out and assembled with MITObim v1.8 (Hahn et al. 2013), which resulted in a complete circular sequence of 151,537 bp in length. Other sequences used in this study were downloaded from the NCBI GenBank database for phylogenetic analysis.

The chloroplast (cp) genome sequence of *V. shenzhenica* (GenBank accession MK962478) was 151,537 bp length and presented a typical quadripartite structure including one large single-copy region (LSC, 87,487 bp), one small single-copy region (SSC, 19,172 bp), and two inverted repeat regions (IRs, 22,439 bp). The cp genome encoded 123 genes, of which 104 were unique genes (69 protein-coding genes, 31 tRNAs, and 4 rRNAs). The overall GC content was 34.96%.

To confirm the phylogenetic position of *V. shenzhenica*, a molecular phylogenetic tree was constructed based on the maximum-likelihood (ML) methods with four species from *Vanilla.* The ML analysis was performed using the CIPRES Science Gateway web server (RAxML-HPC2 on XSEDE 8.2.10) with 1000 bootstrap replicates and settings as described by Stamatakis et al. (2008). The results showed that *V. shenzhenica* is mostly related to taxa with *V. aphylla.* (Figure 1). This newly reported chloroplast genome provides a good foundation for the identification and genotyping of *Vanilla* species.

**Figure 1.**
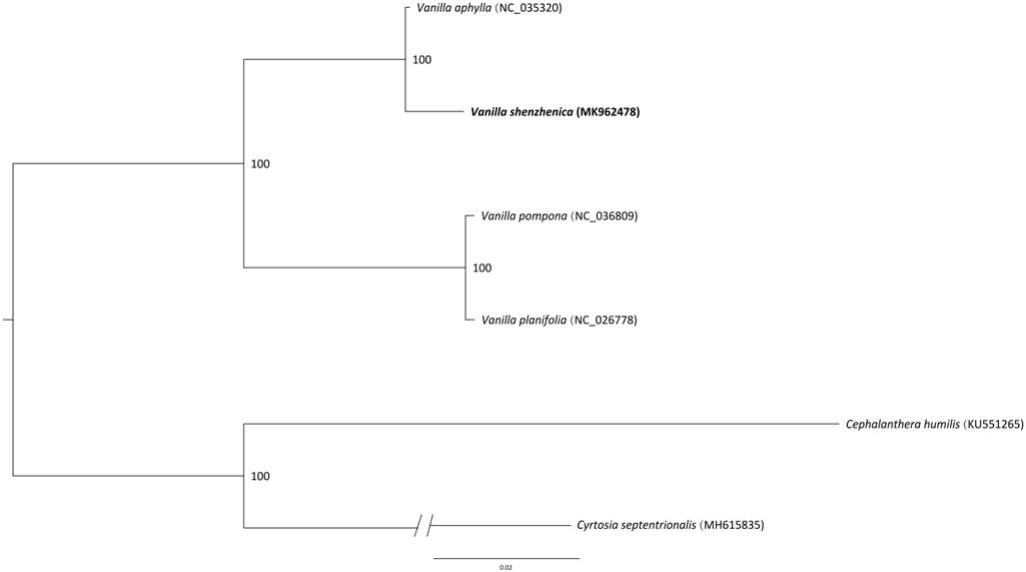
Phylogenetic position of *Vanilla shenzhenica* inferred by maximum-likelihood (ML) of complete cp genome. The bootstrap values are shown next to the nodes.
